# Genetic Diversity and Distribution of the Ciguatera-Causing Dinoflagellate *Gambierdiscus* spp. (Dinophyceae) in Coastal Areas of Japan

**DOI:** 10.1371/journal.pone.0060882

**Published:** 2013-04-11

**Authors:** Tomohiro Nishimura, Shinya Sato, Wittaya Tawong, Hiroshi Sakanari, Keita Uehara, Md Mahfuzur Rahman Shah, Shoichiro Suda, Takeshi Yasumoto, Yohsuke Taira, Haruo Yamaguchi, Masao Adachi

**Affiliations:** 1 Faculty of Agriculture, Kochi University, Nankoku, Kochi, Japan; 2 The United Graduate School of Agricultural Sciences, Matsuyama, Ehime University, Ehime, Japan; 3 Royal Botanic Garden Edinburgh, Edinburgh, United Kingdom; 4 Cardiff University, Cardiff, Wales, United Kingdom; 5 Faculty of Science, University of the Ryukyus, Nakagami District, Okinawa, Japan; 6 College of Ocean Science, Jeju National University, Jeju, South Korea; 7 National Research Institute of Fisheries Science, Yokohama, Kanagawa, Japan; 8 Okinawa Institute of Science and Technology Evolutionary Systems Biology Unit, Kunigami District, Okinawa, Japan; University of New South Wales, Australia

## Abstract

**Background:**

The marine epiphytic dinoflagellate genus *Gambierdiscus* produce toxins that cause ciguatera fish poisoning (CFP): one of the most significant seafood-borne illnesses associated with fish consumption worldwide. So far, occurrences of CFP incidents in Japan have been mainly reported in subtropical areas. A previous phylogeographic study of Japanese *Gambierdiscus* revealed the existence of two distinct phylotypes: *Gambierdiscus* sp. type 1 from subtropical and *Gambierdiscus* sp. type 2 from temperate areas. However, details of the genetic diversity and distribution for Japanese *Gambierdiscus* are still unclear, because a comprehensive investigation has not been conducted yet.

**Methods/Principal Finding:**

A total of 248 strains were examined from samples mainly collected from western and southern coastal areas of Japan during 2006–2011. The SSU rDNA, the LSU rDNA D8–D10 and the ITS region were selected as genetic markers and phylogenetic analyses were conducted. The genetic diversity of Japanese *Gambierdiscus* was high since five species/phylotypes were detected: including two reported phylotypes (*Gambierdiscus* sp. type 1 and *Gambierdiscus* sp. type 2), two species of *Gambierdiscus* (*G. australes* and *G*. cf. *yasumotoi*) and a hitherto unreported phylotype *Gambierdiscus* sp. type 3. The distributions of type 3 and *G*. cf. *yasumotoi* were restricted to the temperate and the subtropical area, respectively. On the other hand, type 1, type 2 and *G. australes* occurred from the subtropical to the temperate area, with a tendency that type 1 and *G. australes* were dominant in the subtropical area, whereas type 2 was dominant in the temperate area. By using mouse bioassay, type 1, type 3 and *G. australes* exhibited mouse toxicities.

**Conclusions/Significance:**

This study revealed a surprising diversity of Japanese *Gambierdiscus* and the distribution of five species/phylotypes displayed clear geographical patterns in Japanese coastal areas. The SSU rDNA and the LSU rDNA D8–D10 as genetic markers are recommended for further use.

## Introduction

Ciguatera fish poisoning (CFP) is one of the most significant marine food-borne illnesses caused by eating reef fish, it affects 25,000–500,000 people annually around the world; and this is endemic throughout the tropical and subtropical Pacific, Indian Ocean and the tropical Caribbean Sea [1], [2], [3], [4], [5], [6], [7]. Common symptoms of this syndrome include gastrointestinal, neurological and cardiovascular disturbances [5], [6], [8]. In some cases, the neurological disturbance usually resolves within weeks of onset, although some symptoms related to the nervous system may persist for months or years [5], [8].

Occurrence of CFP incidents was rather rare in Japan. Until the 1980’s CFP incidents were restricted within the subtropical area (Okinawa region) [9]; however, in recent years CFP incidents have increasingly been reported not only from the subtropical area but also from the temperate area (including the main islands of Japan, Honshu, Shikoku and Kyushu regions) [10], [11], [12]. Thus, the public health problem is becoming more serious along Japanese coastal areas.

In the late 1970’s, a marine epiphytic dinoflagellate was discovered and identified as a possible producer of the toxins responsible for CFP [13] and later described as *Gambierdiscus toxicus* Adachi and Fukuyo by morphological observations [14]. *G. toxicus* could produce ciguatoxins (CTXs) which are transferred to herbivorous and carnivorous fish via food chain [13]. The genus *Gambierdiscus* may also produce other toxins such as maitotoxins (MTXs), gambierol and gambieric acid [15].

Routine identification of *Gambierdiscus* species using light microscopy (LM) and scanning electron microscopy (SEM) is very difficult even for trained taxonomists because species descriptions are based on subtle differences in the thecal plate morphology [14], [15], [16], [17], [18], [19], [20] that are mostly quantitative and largely overlap. Some early studies described three species of *Gambierdiscus* based on SEM observations, that is, *G. toxicus* [14], *G. belizeanus* [16], *G. yasumotoi* [17].

Molecular information has enabled us to assess the phylogenetic relationship more objectively, and *Gambierdiscus* species are by no means excluded from this technical advance [e.g. [20]]. All the phylogenetic analyses of *Gambierdiscus* conducted so far have been inferred from nuclear-encoded ribosomal RNA gene (rDNA) and have shown that each species is partitioned into unique clades that correspond with its morphological features [15], [18], [20]. In 1999, Chinain et al. [18] described *G. pacificus*, *G. australes* and *G. polynesiensis* by morphological and phylogenetic analysis of the D8–D10 region of the nuclear large subunit (LSU) rDNA (LSU rDNA D8–D10) for the first study introducing molecular phylogenetic technique for the classification of *Gambierdiscus* species. In 2009, Litaker et al. [20] described four new species, *G. caribaeus*, *G. carolinianus*, *G. carpenteri* and *G. ruetzleri*, based on morphological characteristics together with molecular phylogenies using the nuclear small subunit (SSU) rDNA, the D1–D3 region of the LSU rDNA (LSU rDNA D1–D3) and the LSU rDNA D8–D10. They also performed a sequence analysis of the ITS1-5.8 S rDNA-ITS2 (ITS region) in order to discriminate *G. ruetzleri* from its sister species *G. yasumotoi* as both species were the most closely related of the *Gambierdiscus* species [20]. Furthermore, an amended description of *G. toxicus* with the designation of an epitype specimen was provided [20]. In their subsequent paper in 2010, two phylotypes (putative species), called *Gambierdiscus* ribotype 1 and *Gambierdiscus* ribotype 2, were reported based on the LSU rDNA D8–D10 phylogeny from the Atlantic [21]. Coincidently in 2010 from Japanese coastal areas in the Pacific, a further two putative allopatric phylotypes were detected with the SSU rDNA and the ITS region: *Gambierdiscus* sp. type 1 from the subtropical and *Gambierdiscus* sp. type 2 from the temperate areas [22]. Recently in 2011, Fraga et al. [15] described *G. excentricus* by morphological and phylogenic analyses of the LSU rDNA D1–D3 and the LSU rDNA D8–D10. In 2012 from Jeju Island, Korea, Pacific, Jeong et al. [23] reported the existence of a possibly cryptic species of ‘*G. caribaeus*’ by using phylogenies of the SSU rDNA, the LSU rDNA D1–D3 and the LSU rDNA D8–D10, and morphological observations by SEM.

*Gambierdiscus* spp. distribute globally from the Atlantic (including Gulf of Mexico and Caribbean Sea) to the Pacific (Australia to Hawaii) [[21], [24] and refs therein] as well as the Bay of Bengal, India [25], Cau Island, Vietnam [26], Indonesia [27], La Union, Philippines [28], Langkawi Island, Redang Island, Port Dickson, Kota Kinabalu and Sipadan Island, Malaysia [29], [30], [31], Jeju Island, Korea [23], [32] and Taiwan [33]. Japanese coasts have also been investigated over the past few decades and revealed that *Gambierdiscus* spp. distribute widely from subtropical to temperate areas [11], [22], [34], [35], [36], [37], [38]. Additionally, in previous studies in Japan, ‘*G. toxicus*’ has been found from the temperate to subtropical areas in Japanese coasts based on morphologically observations [11], [34], [35], [36], [37].

In the present study, we apply the phylogeographic approach with the genetic marker used by Litaker et al. [20] (the SSU rDNA, the LSU rDNA D8–D10 and the ITS region) to have a more complete picture of the diversity of Japanese *Gambierdiscus* spp. We particularly placed emphasis on revealing the distribution of each species and each phylotype, which we define here as a monophyletic entity based on a molecular phylogeny that has not been taxonomically described but has accumulated an equivalent degree of sequence divergence with other described species. In addition, toxicity was tested with mouse bioassay for a representative clone selected from each phylotype.

## Results

### Phylogenetic Analyses of the SSU and the LSU D8–D10

**Figure 1:**
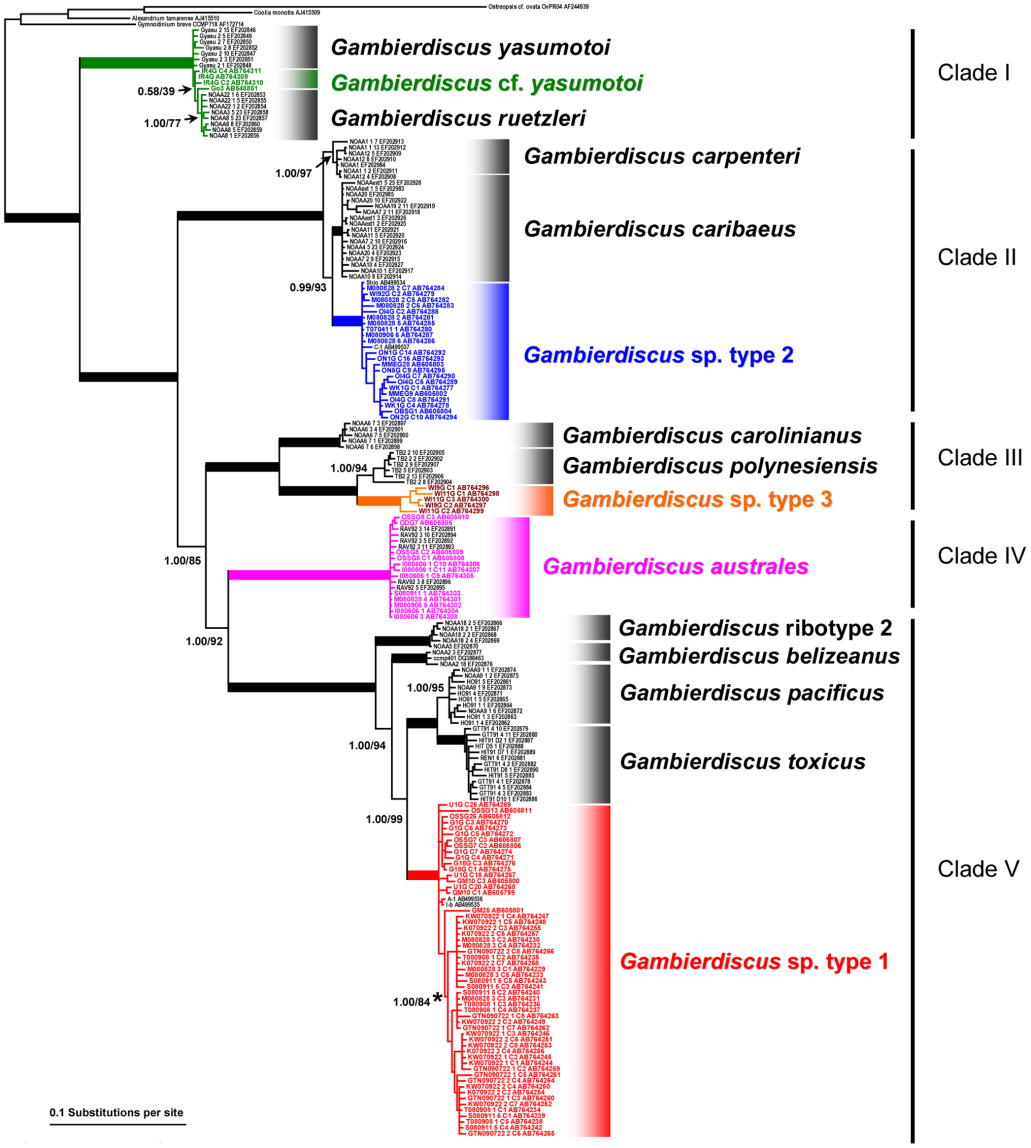
Bayesian inference (BI) phylogeny of the SSU rDNA of *Gambierdiscus* species/phylotypes. Nodal supports are of Bayesian posterior probability (pp) and Bootstrap (bt) values obtained by BI analysis and Maximum likelihood (ML) analysis, respectively. Nodes with strong supports (pp/bt  = 1.00/100) are shown as thick lines. For sequences obtained via cloning, a variant ID, starting with C, is shown followed by strain ID (i.e. KW070922_1_C4). Sequences obtained in the present study are indicated in color. A moderately supported branch (1.00/84) indicated by an asterisk consists of the temperate strains, whereas the others in *Gambierdiscus* sp. type 1 are all of the subtropical origin.

**Figure 2:**
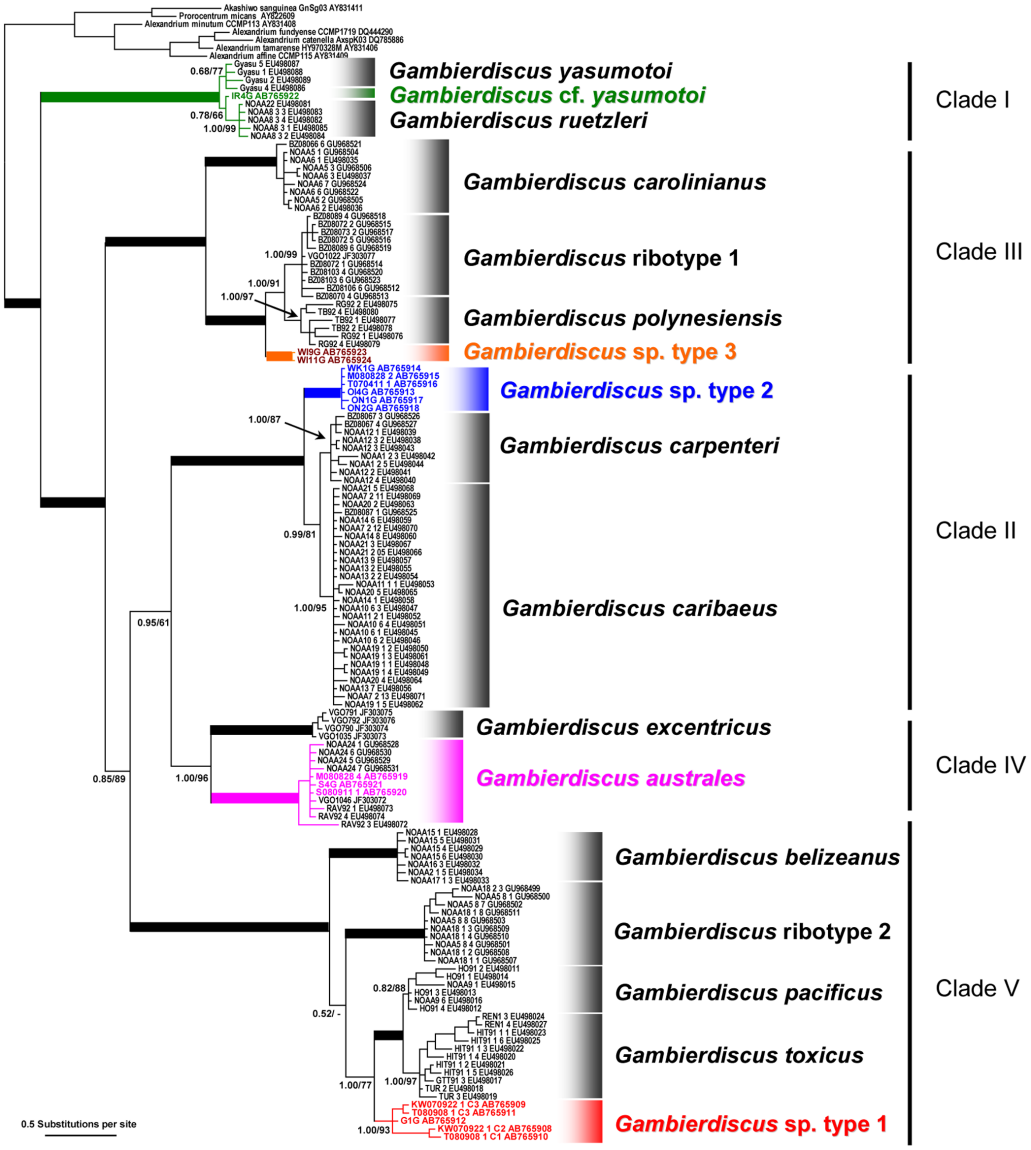
Bayesian inference (BI) phylogeny of the D8–D10 region of the LSU rDNA of *Gambierdiscus* species/phylotypes. Nodal supports are of Bayesian posterior probability (pp) and Bootstrap (bt) values obtained by BI analysis and Maximum likelihood (ML) analysis, respectively. Nodes with strong supports (pp/bt  = 1.00/100) are shown as thick lines. For sequences obtained via cloning, a variant ID, starting with C, is shown followed by strain ID (i.e. T080908_1_C1). Sequences obtained in the present study are indicated in color.

The topologies of Bayesian inference (BI) tree for the SSU rDNA and the LSU rDNA D8–D10 of *Gambierdiscus* spp. including species/phylotypes from Japan (Figs. 1 and 2) were almost the same and the same as that of the Maximum likelihood (ML) tree for the LSU rDNA D8–D10 of *Gambierdiscus* spp. including Japanese species/phylotypes (Fig. S1). The topologies of the former BI trees were almost the same as that of BI tree of *Gambierdiscus* spp. for the SSU rDNA reported by Litaker et al. [20] and those for the LSU rDNA D8–D10 reported by Fraga et al. [15] and Litaker et al. [20], [21]. Discrepancies between the ML and the BI trees were only found in the positions of *Gambierdiscus* ribotype 2 and *G. belizeanus* (Fig. 2 and Fig. S1). Therefore we used the BI trees for discussion of both the SSU rDNA and the LSU rDNA D8–D10 phylogenies.

Both BI phylogenetic trees showed that, each *Gambierdiscus* species/phylotype was clearly separated into five genetically distinct clades I–V and each clade received the highest nodal supports (Figs. 1 and 2), i.e. Bayesian posterior probability (pp)/bootstrap value (bt) = (1.00/100) obtained by BI analysis and ML analysis, respectively. In the SSU rDNA tree (Fig. 1), the major clades I–V branched off one by one in a ladder-like fashion, viz. (I(II(III(IV,V)))). The topology LSU rDNA D8–D10 tree (Fig. 2) was substantially the same as that of the SSU rDNA tree recovering five major clades, except for the position of clade II that was sister to clade IV, viz., (I(III((II,IV)V))).

Clade I was comprised of *G. yasumotoi* (0.68/77 for the LSU rDNA D8–D10 tree), *G. ruetzleri* (1.00/77 and 1.00/99 for the SSU rDNA tree and for the LSU rDNA D8–D10 tree, respectively) and two Japanese strains IR4 G and Go3 (0.58/39 for the SSU rDNA tree, no the LSU rDNA D8–D10 sequence for the latter strain) established in the present study (green letters), the both strains were provisionally named as *Gambierdiscus* cf. *yasumotoi* in the present study based on its gross morphology and sequence analysis of the ITS region (see below, genetic distance section).

Clade II, in the SSU rDNA tree (Fig. 1), had *G. carpenteri* (1.00/97) which diverged at first followed by the second divergence of *G. caribaeus* (1.00/100) and *Gambierdiscus* sp. type 2 (1.00/100), whereas, in the LSU rDNA D8–D10 tree (Fig. 2), the first diverged species/phylotype was *Gambierdiscus* sp. type 2 (1.00/100) and the second ones were *G. carpenteri* (1.00/87) and *G. caribaeus* (1.00/95). Type 2, in the SSU rDNA tree (Fig. 1), was solely comprised of Japanese strains including 14 strains established in the present study (blue letters) and two strains (black letters) by Kuno et al. [22].

Clade III had *G. carolinianus* (1.00/100 and 1.00/100) which was sister to *G. polynesiensis* (1.00/94 and 1.00/97), *Gambierdiscus* ribotype 1 (1.00/99 for the LSU rDNA D8–D10 tree) whose SSU rDNA sequences were unavailable and *Gambierdiscus* sp. type 3 (1.00/100 and 1.00/100) including two strains (WI9 G and WI11 G) established in the present study (orange letters).

Clade IV was exclusively consisted of strains of *G. australes* (1.00/100 and 1.00/100), in the SSU rDNA tree (Fig. 1), among which seven strains were collected in the present study (purple letters) and one strain (black letter) by Litaker et al. [20], and *G. excentricus* (1.00/100) whose SSU rDNA sequences were unavailable in the LSU rDNA D8–D10 tree (Figs. 1 and 2).

Clade V was the most diverse clade, containing five robust phylotypes/species - *Gambierdiscus* ribotype 2 (1.00/100) diverged at first followed by the second divergence of *G. belizeanus* (1.00/100) in the SSU rDNA tree (Fig. 1), whereas the first diverged phylotype was *G. belizeanus* and the second one was *Gambierdiscus* ribotype 2 in the LSU rDNA D8–D10 tree (Fig. 2). Subsequently, *Gambierdiscus* sp. type 1 (1.00/100 and 1.00/93), in the SSU rDNA tree (Fig. 1), contained 16 strains collected in the present study (red letters) and two strains (black letters) by Kuno et al. [22], branched off that was sister to *G. pacificus* (1.00/95 and 0.82/88) and *G. toxicus* (1.00/100 and 1.00/97).

Within type 1, in the SSU rDNA tree, there was a distinct phylogenetic structure corresponding to the geographic origin of the strains, i.e. a moderately supported branch (1.00/84) indicated by an asterisk in Fig. 1 was consisted of the temperate strains, whereas the others in the type 1 were all of subtropical origin.

As a consequence, the genetic diversity of Japanese *Gambierdiscus* revealed in the present study was high since we detected five species/phylotypes including two species of *Gambierdiscus* (*G. australes* and *G*. cf. *yasumotoi*), two reported phylotypes (*Gambierdiscus* sp. type 1 and *Gambierdiscus* sp. type 2 [22]) and a hitherto unreported phylotype *Gambierdiscus* sp. type 3.

### Genetic Distances

For revealing whether Japanese phylotypes (*Gambierdiscus* sp. type 1, type 2 and type 3) had species level divergence or not, we calculated *p* distance of the both genes using uncorrected genetic distance (UGD) model among *Gambierdiscus* species. In case of the SSU rDNA, the mean *p* distance of within-species and that of between-species were 0.003±0.002 and 0.139±0.042, respectively (Table 1). The latter value was significantly different from the former value (*t*-test, *P*<0.01). The minimum *p* distance of between-species (0.004) was found between *G. yasumotoi* and *G. ruetzleri* (Table 1 and Table S1). For the LSU rDNA D8–D10, the mean *p* distance of within-species and between-species were 0.002±0.002 and 0.121±0.036, respectively (Table S2). Between-species divergence of the LSU rDNA D8–D10 was significantly different from the within-species divergence (*t*-test, *P<*0.01). The minimum *p* distance of between-species (0.007) was found between *G. yasumotoi* and *G. ruetzleri* (Tables S2 and S3). The mean *p* distance of between-species of the SSU rDNA was significantly different from that of the LSU rDNA D8–D10 (*t*-test, *P*<0.05).

**Table 1:** Summary of genetic distances of the SSU rDNA over the p distance calculated by uncorrected genetic distance (UGD) model among and within 66 consensus sequences ( = 66 strains; each consensus sequence was constructed with cloned sequences and/or directly sequence of each strain) of *Gambierdiscus* species/phylotypes, and other protists.

Protist	Genus		bp*	n**	Min.	Max.	Mean	SD	Reference***
Dinoflagellate	*Gambierdiscus*	within-species	1759	10	0.000	0.005	0.003	0.002	This study
		between-species	1759	10	0.004	0.180	0.139	0.042	This study
		type 1 vs. another species	1759	11	0.040	0.172	0.121	0.050	This study
		type 2 vs. another species	1759	11	0.028	0.163	0.129	0.051	This study
		type 3 vs. another species	1759	11	0.033	0.168	0.140	0.042	This study
Dinoflagellate	*Peridinium*		1687	9	0.013	0.083	0.055	0.021	Recalculated after Ki et al., 2011 [40]
			1687	9	0.015	0.085	NC****	NC****	Ki et al., 2011 [40]
Diatom	*Cyclotella*		1704	6	0.004	0.034	0.019	0.009	Recalculated after Jung et al., 2010 [41]
			1704	6	0.005	0.035	NC****	NC****	Jung et al., 2010 [41]
	*Discostella*		1704	4	0.010	0.015	0.013	0.002	Recalculated after Jung et al., 2010 [41]
			1704	4	0.011	0.017	NC****	NC****	Jung et al., 2010 [41]
Diatom	2 orders*****		c. 1600		0.01	0.04	0.01	NC****	Moniz and Kaczmarska, 2009 [42]

bp*:Nucleotide bases of the SSU rDNA used for calculations.

n**: Numbers of species (species/phylotypes) used for calculations.

Reference***: Each ref. calculated molecular similarity (we converted values to uncorrected *p* distance) of individual SSU rDNA.

NC****: Not calculated.

2 orders*****: The values include between-species data set for 2 orders; Thalassiosirales and Naviculales.

We calculated the minimum *p* distances of the two genes between each Japanese phylotype (type 1, type 2 and type 3) and another species. Then, we compared those of the minimum values and the minimum value obtained from between-species. As a result, we revealed that the minimum values obtained between each Japanese phylotype and another species, e.g. type 1 vs. *G. pacificus* (0.040 and 0.018 for the SSU rDNA and the LSU rDNA D8–D10, respectively), type 2 vs. *G. caribaeus* (0.028 for the SSU rDNA), type 2 vs. *G. carpenteri* (0.025 for the LSU rDNA D8–D10) and type 3 vs. *G. polynesiensis* (0.033 and 0.024), were larger than that of the pair *G. yasumotoi* vs. *G. ruetzleri* (0.004 and 0.007), which was the minimum value of between-species (Table 1 and Table S2).

The SSU rDNA and the LSU rDNA D8–D10 phylogenies indicated that our strains IR4G and Go3 (LSU rDNA D8–D10 sequence was unavailable for the latter strain) belonged to the clade I along with *G. yasumotoi* and *G. ruetzleri* (Figs. 1 and 2). A further test of their within-clade relationship by means of the uncorrected *p* distance of the ITS region among the strain IR4G (GenBank accession number: AB765925), *G. yasumotoi* and *G. ruetzleri* showed that IR4G was more closely related to *G. yasumotoi* than *G. ruetzleri*, i.e. the *p* distance between IR4G and *G. yasumotoi* was 0.0252 (12 sites in 476 bp), whereas between IR4G and *G. ruetzleri* was 0.0479 (23 sites in 480 bp). Therefore, Japanese 2 strains (IR4G and Go3) were tentatively identified as *G*. cf. *yasumotoi* by referring to the indicative *p* distance of the species limit on the ITS region according to Litaker et al. [39].

### Distribution of *Gambierdiscus* in Coastal Areas of Japan

**Figure 3:**
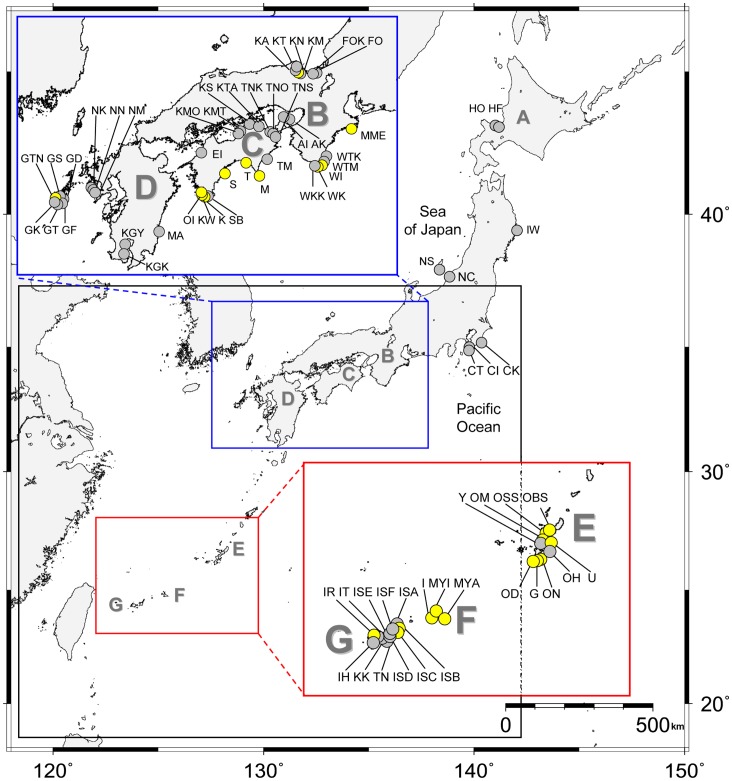
Map of research area. Location of each sampling station and its ID as well as presence (yellow circle) or absence (gray circle) of *Gambierdiscus* cells in the sample is shown. Each island is marked as A: Hokkaido, B: Honshu (main Isl.), C: Shikoku, D: Kyushu, E: Okinawa Island, F: Miyako Island, Ikema Island and Irabu Island (from right to left), G: Ishigaki Island and Iriomote Island (from right to left). A: the boreal area, B, C and D: the temperate area, E, F and G: the subtropical area.

**Figure 4:**
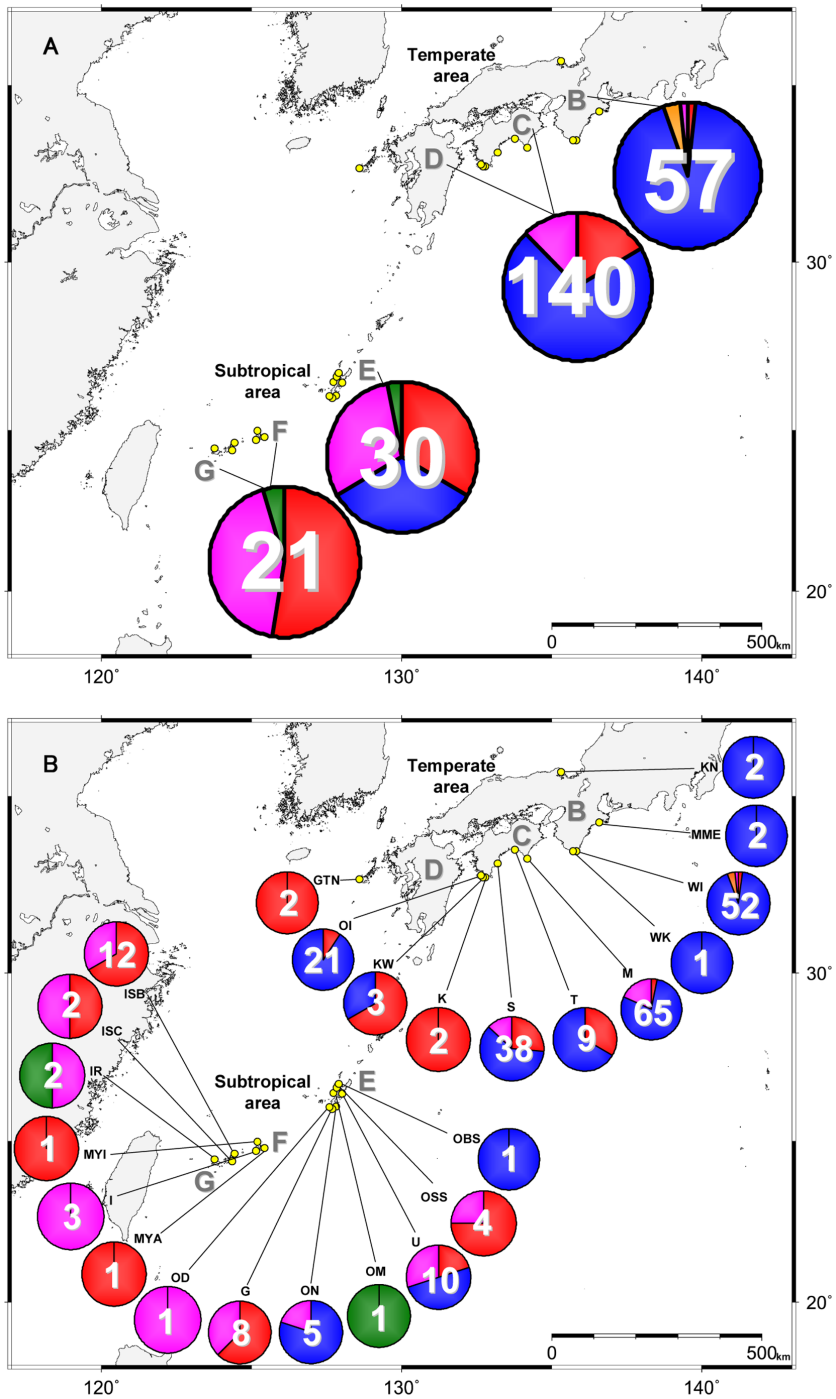
Geographic distribution of *Gambierdiscus* species/phylotypes. Enlargement of a part enclosed by an open square in Fig. 3. The numbers in each pie indicate the number of strains used for phylotyping. Each color in pies corresponds to a species/phylotype in phylogenetic trees in Figs. 1 and 2, i.e. red: *Gambierdiscus* sp. type 1, blue: *Gambierdiscus* sp. type 2, orange: *Gambierdiscus* sp. type 3, purple: *G. australes*, green: *G*. cf. *yasumotoi*. Each island is marked as B: Honshu (main Isl.), C: Shikoku, D: Kyushu, E: Okinawa Island, F: Miyako Island, Ikema Island and Irabu Island (from right to left), G: Ishigaki Island and Iriomote Island (from right to left). B, C and D: the temperate area, E, F and G: the subtropical area. Figure 4A: Total number of strains and composition of each species/phylotype. Figure 4B: Breakdown of species/phylotype composition from each sampling station.

We collected macroalgal samples from 73 stations (50 and 23 originated from the temperate area and the subtropical area, respectively) in Japan during 2006–2011 (Fig. 3 and Table S4). In total 248 unialgal strains of *Gambierdiscus* (197 and 51 originated from the temperate area and the subtropical area, respectively) were successfully established from 24 stations (11 and 13 originated from the temperate area and the subtropical area, respectively) (yellow circles in Fig. 3). In the present study, five *Gambierdiscus* species/phylotypes (*G. australes*, *G.* cf. *yasumotoi*, *Gambierdiscus* sp. type 1, *Gambierdiscus* sp. type 2 and *Gambierdiscus* sp. type 3) were found in the western and the southern coastal areas of Japan (areas B, C, D, E, F and G in Figs. 3 and 4). On the other hand, our 248 strains contained no *G. toxicus.* No *Gambierdiscus* cell was found in samples collected from all stations of the Sea of Japan, except for KN, and some stations along Pacific coast (gray circles in Fig. 3). Details of strains are summarized in Table S5.

The species/phylotype composition plotted onto a map showed a species/phylotype-specific pattern of their distribution (Fig. 4). For example, *G.* cf. *yasumotoi* and type 3 were restricted in the subtropical area (from the station OM and IR in areas E and G, respectively) and the temperate area (WI in area B), respectively (Fig. 4B). On the other hand, type 1, type 2 and *G. australes* were widespread from the temperate area (areas B, C and D in Fig. 4) to the subtropical area (areas E, F and G in Fig. 4) in the areas studied (Table 2 and Table S4), with a tendency that type 1 and *G. australes* were dominant in the subtropical area, whereas type 2 was more abundant in the temperate area (Fig. 4A). Although type 2 exhibited a widespread distribution (Table 2, Fig. 4), they were totally absent at the southern end of our study field (subtropical island areas F and G in Fig. 4). Each sampling station usually held 1–3 species/phylotype (s), with an exceptional case in that four species/phylotypes (type 1, type 2, type 3 and *G. australes*) were recorded by during samplings on two different dates at a temperate station WI (area B in Fig. 4B).

**Table 2:** Details of sampling stations where each *Gambierdiscus* species/phylotype was observed.

Species/phylotype	SWT (°C)	Salinity	Latitude (sampling station)	Area	Station
*Gambierdiscus* sp. type 1	21.1–32.4	29.2–33.0	24°26′40 N (ISC)–33°27′10 N (WI)	B, C, D, E, F, G	WI, M, T, S, K, KW, OI, GTN, OSS, U, G, MYA, MYI, ISC, ISB
*Gambierdiscus* sp. type 2	17.2–30.0	29.7–33.2	26°07′58 N (ON)–32°52′32 N (KN)	B, C, E	KN, MME, WI, WK, M, T, S, KW, OI, OBS, U, ON
*Gambierdiscus* sp. type 3	22.2	ND*	33°27′10 N (WI)	B	WI
*Gambierdiscus australes*	19.1–32.4	30.5–32.2	24°26′40 N (ISC)–33°27′10 N (WI)	B, C, E, F, G	WI, M, S, OSS, U, ON, G, OD, I, IR, ISC, ISB
*Gambierdiscus* cf. *yasumotoi*	24.8	ND*	24°25′06 N (IR)–26°26′41 N (OM)	E, G	OM, IR

ND*: No data.

We compared the occurrence of each species/phylotype with sea water temperature (SWT) measured when the samples were taken. Tendencies that type 1 and *G. australes* were more abundant in warmer water and that type 2 more commonly occurred in cooler water were found (Table 2), e.g. type 1 cells were isolated from 21.1°C to 32.4°C, *G. australes* cells were isolated from 19.1°C to 32.4°C, whereas type 2 cells were isolated from 17.2°C to 30.0°C (Table 2). We found no tendency of the occurrence of each species/phylotype with salinity (Table 2).

### Toxicity Analyses

The toxicities in mice were tested by means of the dichloromethane soluble fraction (DSF, a fraction expected to contain CTXs) and aqueous methanol soluble fraction (MSF, a fraction expected to contain MTXs) using six strains representing four species/phylotypes, viz., KW070922_1 (*Gambierdiscus* sp. type 1); M080828_2 and T070411_1 (*Gambierdiscus* sp. type 2); WI9G and WI11G (*Gambierdiscus* sp. type 3); S080911_1 (*G. australes*). The results of mouse bioassay indicated the presence of toxicity in type 1, type 3 and *G. australes* but not in type 2 (Table 3 and Table S6). In DSF toxicity, *G. australes* was the highest (670×10^−4^ MU 1,000 cells^−1^) and type 1 also showed positive result (20 × 10^−4^ MU 1,000 cells^−1^). In MSF toxicity, both type 1 and *G. australes* showed positive result (67×10^−4^ MU 1,000 cells^−1^). Although one strain of type 3 (WI11G) was positive for MSF toxicity (67 MU 1,000 cells^−1^), another strain WI9G was negative (Table 3 and Table S6).

**Table 3:** Toxicities of Japanese *Gambierdiscus* species/phylotypes tested by a mouse bioassay. One MU is defined as the LD_50_ dose for a 20-g mouse over 24 h.

Species/phylotype	Strain	DSF toxicity*	MSF toxicity**	Reference
		(× 10^−4 ^MU/1,000 cells)	(× 10^−4 ^MU/1,000 cells)	
*Gambierdiscus* sp. type 1	KW070922_1	20	67	This study
*Gambierdiscus* sp. type 2	M080828_2	―^***^	―^***^	This study
	T070411_1	―^***^	ND^****^	This study
*Gambierdiscus* sp. type 3	WI9G	―^***^	―^***^	This study
	WI11G	―^***^	67	This study
*Gambierdiscus australes*	S080911_1	670	67	This study
	RAV-92	4	0.2	Chinain et al., 1999 [18]
*Gambierdiscus toxicus*	GTT-91	―^***^	0.7	Chinain et al., 1999 [18]
	REN-1	―^***^	1.7	Chinain et al., 1999 [18]
	TUR	―^***^	0.6	Chinain et al., 1999 [18]
*Gambierdiscus pacificus*	HO-91	9	0.7	Chinain et al., 1999 [18]
*Gambierdiscus polynesiensis*	TB-92	1500	0.1	Chinain et al., 1999 [18]
	RG-92	800	0.06	Chinain et al., 1999 [18]

DSF*: Dichloromethane soluble fraction (DSF) toxicities correspond to CTXs toxicity.

MSF**: Aqueous methanol soluble fractions (MSF) toxicities correspond to MTXs toxicity.

―***: Not detected.

ND****: Not done.

## Discussion

### Genetic Diversity of Japanese *Gambierdiscus*

A previous phylogeographic study of Japanese *Gambierdiscus* using the SSU rDNA and the ITS region revealed the existence of two putative allopatric phylotypes: *Gambierdiscus* sp. type 1 and *Gambierdiscus* sp. type 2 [22]. On the other hand, we detected five species/phylotypes including the two reported phylotypes of *Gambierdiscus* (type 1 and type 2 [22]), two species of *Gambierdiscus* (*G. australes* and *G*. cf. *yasumotoi*) and a hitherto unreported phylotype *Gambierdiscus* sp. type 3 from western and southern coastal areas of Japan. Considering these results, the unexpectedly high genetic diversity of Japanese *Gambierdiscus*, up to five species/phylotypes in the present study, might simply be explained by the intensiveness of sampling, viz. the more strains established the more probability to encounter something new.

The minimum uncorrected *p* distances (0.028–0.040) of the SSU rDNA obtained from the pairs between Japanese phylotypes (type 1, type 2 and type 3) and another species, were larger than that of the pair *G. yasumotoi* vs. *G. ruetzleri* (0.004) which was the minimum value of between-species of *Gambierdiscus*. Furthermore, these values were also larger than the minimum *p* distances calculated for between-species from other protists, e.g. dinoflagellate *Peridinium* (between 9 species, 0.013) [40], diatom *Cyclotella* (between 6 species, 0.004) [41], diatom *Discostella* (between 4 species, 0.010) [41] and 2 orders of diatoms (0.01) [42] (Table 1). Therefore, the minimum *p* distances obtained from the pairs between Japanese phylotypes and another species are most likely indicative of species level divergence, and Japanese phylotypes will deserve to be described as new species. Currently detailed morphological investigations are ongoing to support the description of these phylotypes (Nishimura et al. unpubl.). It also should be noted that the minimum *p* distances obtained from the pairs *G. yasumotoi* vs. *G. ruetzleri* was the same as or lower than the values obtained from other protists, and was close to those within-species level divergence of *Gambierdiscus* species.

The mean *p* distance of the SSU rDNA obtained from between-species (0.139) was larger than those of other protists, e.g. dinoflagellate *Peridinium* (between 9 species, 0.055) [40], diatom *Cyclotella* (between 6 species, 0.019) [41], diatom *Discostella* (between 4 species, 0.013) [41] and 2 orders of diatoms (0.01) [42]. These results suggest that the genetic diversity of *Gambierdiscus* seems to be larger than other protists.

In relation to *Gambierdiscus* sp. type 2 from Japanese coastal waters, *G. caribaeus* GCJJ1 strain from Jeju Island, Korea, Pacific was reported to be closely related with a Japanese strain C-1, which belongs to *Gambierdiscus* sp. type 2 [22], by Jeong et al. [23]. They also reported that the SSU rDNA sequence of Korean strain GCJJ1, which has not been opened on DNA Data Bank of Japan (DDBJ) yet, was 0.5% different (0.005 for uncorrected *p* distance in the present study) from that of strain C-1 of *Gambierdiscus* sp. type 2, whereas the Korean strain GCJJ1 was 2.4–4.0% different (0.024–0.040 for uncorrected *p* distance) from those of *G. caribaeus* [23]. On the other hand, morphological observations using SEM suggested that morphology of Korean strain GCJJ1 was similar to those of Belize strains of *G. caribaeus*. Considering the similar morphology and the molecular divergence between them, the authors suggested that the Korean strain GCJJ1 may be a cryptic species of *G. caribaeus*. Together with our results that the mean *p* distance (0.139±0.042) and the minimum *p* distance (0.004) among *Gambierdiscus* species, the Korean strain GCJJ1 may belong to *Gambierdiscus* sp. type 2 rather than *G. caribaeus* and the strain as well as Japanese strains of type 2 are to be described as a new species.

The SSU rDNA and the LSU rDNA D8–D10 phylogenies indicated that our strains IR4G and Go3 (LSU rDNA D8–D10 sequence was unavailable for the latter strain) belonged to the clade I, along with *G. yasumotoi* and *G. ruetzleri*. An empirical study by Litaker et al. [39] proposed that uncorrected *p* distance between 0.042 and 0.580 substitutions per site in the ITS region are indicative of species level divergence of the ITS region based on their observations among 14 genera of dinoflagellate. Recently Litaker et al. [20] applied the idea with *Gambierdiscus*, where two morphologically similar but barely and genetically distinguishable species *G. yasumotoi* and *G. ruetzleri* had *p* = 0.07 for the ITS region. As a result of our sequence analyses, we provisionally called our strain IR4G as *G*. cf. *yasumotoi*, given the *p* distance of the ITS region between IR4G and *G. yasumotoi* was 0.0252, whereas between IR4G and *G. ruetzleri* was 0.0479. In the case of the SSU rDNA sequences comparison, however, relationships within clade I are still confusing, because the *p* distances obtained from the pairs *G. yasumotoi* vs. *G. ruetzleri* was lower value comparing with those of other protists, and the value was similar with within-species level divergence of *Gambierdiscus* species. This indicates that additional works are needed to confirm its taxonomical status.

### Distribution of *Gambierdiscus* in Coastal Areas of Japan

Kuno et al. [22] revealed the existence of two allopatric phylotypes: *Gambierdiscus* sp. type 1 from the subtropical area and *Gambierdiscus* sp. type 2 from the temperate area. On the other hand, we confirmed both of the two phylotypes occur from the subtropical area to the temperate area. In addition to those distributions, we found that *G. australes* occurs from the subtropical area to the temperate area, whereas, *G*. cf. *yasumotoi* and *Gambierdiscus* sp. type 3 were restricted to the subtropical area and the temperate area, respectively. Additionally, we found a tendency that type 1 and *G. australes* were dominant in the subtropical area, whereas type 2 was dominant in the temperate area. Considering those trends of the distribution of each species/phylotype in Japan, different growth response of each species/phylotype to various environmental parameters, such as SWT and salinity so on, would determine those trends of the distribution. Comparing those distributions with SWT when each cell was isolated, type 1 and *G. australes* cells were isolated from warmer waters, while type 2 cells were isolated from cooler waters. Additionally, type 2 cells were isolated from KN (35°41′31 N, 135°18′16 E), in which Hatayama et al. [38] has also observed *Gambierdiscus* cells. The sampling station is the most northerly location in the world where *Gambierdiscus* spp. have been reported so far. These results indicate that type 2 appears to prefer cooler waters to warmer waters because of their growth response which probably plays a crucial role in their predomination around the temperate areas in Japan. To confirm this hypothesis, comparative culture experiments with type 1, type 2, type 3 and *G. australes* are currently ongoing (Yoshimatsu et al. unpubl.). Additionally, we found no *Gambierdiscus* cell from Japanese northern stations (HO, HF, IW, NS and NC), where the number of samples was poorly represented in our collection. The absence of *Gambierdiscus* from our northern samples could also be explained by their restricted distribution in warm waters: so far there is no *Gambierdiscus* found from boreal area.

### Toxicity

Chinain et al. [18] applied mouse bioassay (MBA) for detection of toxicities of four *Gambierdiscus* species (*G. toxicus*, *G. pacificus*, *G. polynesiensis* and *G. australes*) isolated from French Polynesia, Pacific and revealed that *G. pacificus*, *G. polynesiensis* and *G. australes* showed both toxicities of DSF (a fraction expected to contain CTXs) and MSF (a fraction expected to contain MTXs), whereas *G. toxicus* showed only MSF toxicity. In the present study, MBA results of Japanese *Gambierdiscus* revealed that three species/phylotypes had toxicities (*G. australes*, *Gambierdiscus* sp. type 1 and *Gambierdiscus* sp. type 3) and a phylotype (*Gambierdiscus* sp. type 2) was thought to be non-toxic. Unfortunately, *G.* cf. *yasumotoi* could not be assessed for its toxicity, because mass culturing was not successful. However a strain of *G. yasumotoi* isolated from Singapore was found to be potentially toxic [17].

In DSF toxicities among Japanese *Gambierdiscus*, the toxicity of *G. australes* was stronger than that of type 1. Additionally, for type 3 which was revealed to be “non-toxic” for DSF in the present study, a strain WI9G exhibited progressive paralysis of mice or death of mice however the proportion that died did not exceed 50%. The DSF toxicity of Japanese *G. australes* was approx. 170 fold stronger than that of *G. australes* isolated from French Polynesia [18]. This suggests that the toxicity of a *Gambierdiscus* species is possibly variable being related with location where each strain was isolated. Furthermore, the degree of DSF toxicity of Japanese *G. australes* followed the toxicities of *G. polynesiensis* strains, which are known as ‘super-toxic strains [43]’. The DSF toxicity of type 1 followed that of Japanese *G. australes*. In MSF toxicities among Japanese *Gambierdiscus*, the toxicities of *G. australes*, type 1 and type 3 were the same value, furthermore those toxicities were stronger than those of *G. toxicus*, *G. pacificus*, *G. polynesiensis* and *G. australes* isolated from French Polynesia [18]. Considering these results, it was revealed that species/phylotypes which have relatively strong DSF and MSF toxicities so far may distribute around Japanese coastal areas.

### Conclusion/Future Prospects

As a result of phylogenies of the SSU rDNA and the LSU rDNA D8–D10, we revealed that five species/phylotypes including two reported phylotypes (*Gambierdiscus* sp. type 1 and *Gambierdiscus* sp. type 2 [22]), two species of *Gambierdiscus* (*G. australes* and *G*. cf. *yasumotoi*) and a hitherto unreported phylotype *Gambierdiscus* sp. type 3 distribute around Japanese coastal areas, especially western and southern areas. Out of Japanese five species/phylotypes, the three phylotypes (type 1, type 2 and type 3) that showed species level genetic divergence by calculating uncorrected genetic distance using these genes will deserve to be described as new species. Also the distribution of Japanese *Gambierdiscus* was revealed; *G*. cf. *yasumotoi* and type 3 distributed allopatrically to the subtropical area and the temperate area, respectively. On the other hand, type 1, type 2 and *G. australes* occurred from the subtropical area to the temperate area, with a tendency that type 1 and *G. australes* were dominant in the subtropical area, whereas type 2 was dominant in the temperate area. We revealed a surprising diversity of Japanese *Gambierdiscus*, and the distribution of five species/phylotypes displayed clear geographical patterns in the subtropical and temperate areas. For revealing toxicities of those species/phylotypes, MBA was conducted and revealed that *G. australes* and type 1 had toxicities of DSF and MSF, and type 3 had only toxicity of MSF, whereas no toxicities of both fractions were detected from type 2. In the DSF toxicities, *G. australes* was stronger than that of type 1. So far, occurrences of CFP incidents have been mainly reported from the subtropical area of Japan that might be explained by the result obtained in the present study that the toxic type 1 and *G. australes* are dominant in subtropical area, viz. it is suggests that one of agents of CFP incidents occurred in the subtropical area of Japan might be *Gambierdiscus* sp. type 1 and/or *G. australes*. Comparative LC-MS studies on extracts from both cultured *Gambierdiscus* and ciguateric fish are required to elucidate as to whether the occurrence of CFP is linked to the toxins produced by *Gambierdiscus*.

As a whole, the SSU rDNA and the LSU rDNA D8–D10 as genetic marker is recommended for further use, i.e. species/phylotype-level comparison such as molecular systematic/taxonomy within the genus. Now we are trying to establish the molecular-based monitoring system using quantitative polymerase chain reaction (qPCR) assay for the detection of each toxic species/phylotype of *Gambierdiscus* in Japanese coastal areas.

## Materials and Methods

### Ethics Statement

No specific permits were required for the described field studies. No specific permission was required for any locations and activity. The locations are not privately owned or protected in any way. No activity during field study involved any endangered species or protected species.

The Animal Use Protocol (AUP) for handling mouse described here was approved by the Animal Ethics Committee (approval ID: D-00068) of Kochi University.

### Isolation and Establishment of *Gambierdiscus* Strains

Macroalgal substrata were collected mainly from the southern part of Japan between 2006 and 2011. Details of the sampling stations are shown in Table S4. In the laboratory the macroalgae were placed in a plastic bottle and vigorously shaken to cause epiphytes, including *Gambierdiscus*, to detach from the substrata. The resultant suspension was sieved twice, firstly through 150 µm and then through 20 µm Nitex meshes. Materials retained on the second mesh filter were resuspended in filtered seawater. *Gambierdiscus* cells placed in a 6 well/flat bottom microplate (Asahi Glass, Tokyo, Japan) were isolated under an inverted microscope referring to the morphological criteria of the genus *Gambierdiscus* described by Adachi and Fukuyo [14]. All the clonal strains were maintained with Daigo IMK (Nihon Pharmaceutical, Tokyo, Japan), IMK/2, IMK/4 or soil-extract-added IMK/4 medium using GF/F-filtered sea water (salinity of 31±1) in polypropylene (PP)-capped test tubes (25×150 mm) with a flat bottom (Maruemu, Osaka, Japan) containing 20 ml medium at 25°C, with 100 µmol photons·m^–2^·s^–1^ from cool-white tubes; the photoperiod was 12∶12 h L:D.

### DNA Extraction, PCR, Cloning and Sequence

To amplify the SSU rDNA and the LSU rDNA D8–D10 we performed direct PCR, which used intact cells for PCR template instead of genomic DNA allowing us to skip the DNA extraction process, in order to analyze large numbers of isolates quicker and more efficiently. For harvesting cells for template, 1 ml of medium containing *Gambierdiscus* cells was centrifuged to make a pellet, which was subsequently washed twice with sterile water. A small fraction was picked from the pellet by a micropipette and transferred into a PCR tube that contained a 25-µl mixture as below. In case direct PCR failed to amplify the SSU rDNA and/or the LSU rDNA D8–D10, and the ITS region for IR4G, genomic DNA was extracted from the strains using DNeasy Plant Mini Kit (Qiagen, Valencia, CA, USA).

The SSU rDNA was amplified by using 50 µM of oligonucleotide primers Dino5’UF [44] and 18 ScomR1 [45] (Table 4). PCR reactions typically contained a 25-µl mixture: 0.5 µl of MightyAmp DNA Polymerase (1.25 U/µl, Takara Bio, Shiga, Japan); primers as above (7.5 pmol each); 12.5 µl of 2×MightyAmp buffer (Mg^2+^, dNTP plus) which contains magnesium chloride (4 mM) and dNTPs (800 µM each). The PCR cycling comprised of an initial 2 min heating step at 98°C, followed by 35 cycles: 98°C for 10 sec, 68°C for 110 sec, and a final extension at 68°C for 5 min. The PCR amplification of the SSU rDNA for strains from several sampling stations (MME, OBS, OSS, OM, OD, MYA and MYI, Table S4) was performed at University of the Ryukyus using primers and methods described in Takano and Horiguchi [46]. The LSU rDNA was amplified by using 50 µM of oligonucleotide primers FD8 and RB [18] (Table 4). The PCR cycling comprised of an initial 2 min heating step at 98°C, followed by 25 cycles: 98°C for 10 sec, 55°C for 15 sec and 68°C for 40 sec, and a final extension at 68°C for 5 min. The ITS region was amplified by using 50 µM of oligonucleotide primers ITSA [47] and D2C [48] (Table 4). The PCR cycling comprised of an initial 2 min heating step at 98°C, followed by 35 cycles: 98°C for 10 sec, 55°C for 15 sec and 68°C for 75 sec, and a final extension at 68°C for 5 min.

**Table 4:** Oligonucleotide primers used for PCR amplification and DNA sequencing.

Primer name	Synthesis direction	Sequence (5'–3')	Anneals to	Reference
**The ITS1-5.8S-ITS2 rDNA**				
ITS A	Forward	GTA ACA AGG THT CCG TAG GT	22–41*	Sato et al., 2011 [47]
type4ITSICF	Forward for sequencing	TTG TTG GTT TCC CCT CAA	83–102*	This study
inner5.8S_F	Forward for sequencing	AAA TTG CAG AAT YCC GTG AG	306–325*	This study
inner5.8S_R2	Reverse for sequencing	TGA CTC ACG GRA TTC TGC	311–328*	This study
type4ITSICR	Reverse for sequencing	GTC TGC CAG TGT CAC AAT GC	473–492*	This study
ITS B	Reverse for sequencing	AKA TGC TTA ART TCA GCR GG	542–561*	Sato et al., 2011 [47]
D2C	Reverse	CCT TGG TCC GTG TTT CAA GA	714–733**	Scholin et al., 1994 [48]
**The SSU rDNA**				
Dino5'UF	Forward	CAA CCT GGT GAT CCT GCC AGT	1–23***	Litaker et al., 2005 [49]
18ScomF1	Forward for sequencing	GCT TGT CTC AAA GAT TAA GCC TAG C	32–56***	Zhang et al., 2005 [45]
18S_G53F	Forward for sequencing	TGC ATG TCT CAG CTT AAG TG	54–73***	This study
G10'F	Forward for sequencing	TGG AGG GCA AGT CTG GTG	549–566***	This study
18S_G623F	Forward for sequencing	GTT AAA AGG CTC GTA GTT GGA	618–638***	This study
G17'F	Forward for sequencing	ATA CCG TCM TAG TCT TAA CC	1007–1026***	Modified after Litaker et al., 2003 [44]
18S_G1249F	Forward for sequencing	GGA TTG ACA GAT TGA CAG CT	1232–1251***	This study
G18F	Forward for sequencing	CAA TAA CAG GTC TGT GAT GC	1426–1445***	Litaker et al., 2003 [44]
18S_G371R	Reverse for sequencing	ACC CTC ATC CTC CGT CAC CT	359–378***	This study
G10R	Reverse for sequencing	CCG CGG CTG CTG GCA CCA GAC	559–579***	Litaker et al., 2005 [49]
18S_G781R	Reverse for sequencing	AAA CAC CTG CTT TGA ACA C	768–786***	This study
G17'R	Reverse for sequencing	GTT TAT GGT TAA GAC TAK GAC GG	1010–1032***	This study
18S_G1301R	Reverse for sequencing	CAC TCC ACC AAC TAA GAA CG	1279–1303***	This study
G18R	Reverse for sequencing	GCA TCA CAG ACC TGT TAT TG	1426–1445***	Litaker et al., 2005 [49]
18S_G1781R	Reverse for sequencing	GAA ACC TTG TTA CGA CTT CT	1758–1777***	This study
18ScomR1	Reverse	CAC CTA CGG AAA CCT TGT TAC GAC	1762–1785***	Zhang et al., 2005 [45]
**The D8–D10 region of the LSU rDNA**				
FD8	Forward	GGA TTG GCT CTG AGG GTT GGG	1980–2000**	Chinain et al., 1999 [18]
GLD8_421F	Forward for sequencing	ACA GCC AAG GGA ACG GGC TT	2395–2414**	This study
GLD8_677R	Reverse for sequencing	TGT GCC GCC CCA GCC AAA CT	2645–2664**	This study
RB	Reverse	GAT AGG AAG AGC CGA CAT CGA	2905–2925**	Chinain et al., 1999 [18]
**T-Vector pMD20**				
U19	Forward	GGT TTT CCC AGT CAC GAC G	In the vector	Applied Biosystems
pUCM13R	Reverse	CAG GAA ACA GCT ATG AC	In the vector	Promega

* : Annealing site in the ITS1-5.8S-ITS2 rDNA sequence of *Gambierdiscus yasumotoi* GYASU, GU968498 (Vandersea et al., 2010 Direct Submission).

** : Annealing site in the LSU rDNA sequence of *Prorocentrum micans*, X16108 (Lenaers et al., 1989 [51]).

*** : Annealing site in the SSU rDNA sequence of *Pfiesteria piscicida*, AY245693 (Litaker et al., 2003 [44]).

The quantity and length of products were examined by agarose gel electrophoresis against known standards. Excess primers and dNTPs were removed from PCR product using High Pure PCR Cleanup Micro Kit (Roche, Tokyo, Japan). Each product was sequenced directly or cloned into the T-vector pMD20 (TaKaRa Bio, Shiga, Japan). Clones were screened for inserts by PCR amplification with plasmid primers U19 and pUCM13R (Table 4).

BigDye® Terminator v3.1 Cycle Sequencing Kit (Applied Biosystems Japan, Chiba, Japan) was used for the sequence of the PCR products and the clones. Primers and excess dye-labeled nucleotides were removed using the Performa DTR V3 clean-up system (Edge Biosystems, Gaithersburg, MD).

For easy and quick phylotyping of strains, approximately 500 bp including the most variable 300 bp region of the SSU rDNA [44] were sequenced by internal sequencing primer G10R [49] (Table 4). Sequencing products were run on an ABI PRISM 3100-Avant Genetic Analyzer (Applied Biosystems Japan, Chiba, Japan). Forward and reverse reads were edited and aligned using SeqMan (DNASTAR, Madison, WI). All the information of clonal strains, including locality, water temperature and source sample are listed in Table S5.

### Phylogeny and Sequence Analyses

The SSU rDNA and the LSU rDNA D8–D10 sequences were aligned using ClustalW [50] with publicly available ones retrieved from DNA Data Bank of Japan (DDBJ). In both rDNA datasets, the 5′ and 3′ ends were manually aligned to truncate and refine the both ends. Finally, ambiguously aligned positions were excluded, resulting in 190 taxa/1757 nucleotides (32 bp–1762 bp site of *Pfiesteria piscicida*, AY245693 followed the alignment used in Litaker et al. [44]) and 136 taxa/846 nucleotides (1980 bp–2978 bp site of *Prorocentrum micans*, X16108 followed the alignment used in Lenaers et al. [51]) in the SSU rDNA and the LSU rDNA D8–D10 dataset, respectively.

MrBayes 3.1.2 [52], [53] was used for Bayesian inference (BI) to estimate the posterior probability distribution using Metropolis-Coupled Markov Chain Monte Carlo (MCMCMC) [53]. MCMCMC from a random starting tree were used in this analysis with two independent runs and 1 cold and 3 heated chain with temperature set 0.2. Trees were sampled every 100 th generation. To increase the probability of chain convergence, we sampled at least 10,000 trees after the standard deviation values of the two runs dipped below 0.01 to calculate the posterior probabilities. RAxML-VI-HPC, v7.0.4 [54] was used for Maximum likelihood (ML) analyses. We conducted a rapid Bootstrap analysis and search for the best-scoring ML tree in one single run with -f a option for 100 repeats. MrModeltest 2 [55] was used to determine the most appropriate model of sequence evolution. The best-fit model according to the Akaike Information Criterion (AIC) was GTR+G, and this was used for BI and ML analyses.

With the ITS region we calculated the uncorrected genetic distance (*p*) to consider substitutions, gaps and ambiguous bases for calculations among *G. yasumotoi*, *G. ruetzleri* and Japanese strain IR4G, in order to find the closest relative of these strains. The ITS region sequences of *G. yasumotoi* and *G. ruetzleri* were obtained from Fig. 74 in Litaker et al. [20]. A consensus of the ITS region sequence for six separate clones (sequences obtained via cloning) of IR4G was used for the analysis to reduce any bias due to the inclusion of pseudogene sequences. Additionally we also calculated *p* distance using uncorrected genetic distance (UGD) model (*p*-distance model in MEGA 5.05 [56]) among *Gambierdiscus* species in that strict consensus sequences were obtained both for each species using the SSU rDNA and the LSU rDNA D8–D10 and revealed the *p* distances of within-species and between-species. Then, we calculated the *p* distances between each Japanese phylotype and another species. All positions containing alignment gaps and missing data were eliminated only in pairwise comparisons (Pairwise deletion option in MEGA 5.05 [56]). For the UGD model of the SSU rDNA and the LSU rDNA D8–D10 calculation in the present study, using MEGA 5.05, gaps and ambiguous bases were not considered.

### Toxicity Analyses

Six strains representing four species/phylotypes, viz., KW070922_1 (*Gambierdiscus* sp. type 1); M080828_2 and T070411_1 (*Gambierdiscus* sp. type 2); WI9G and WI11G (*Gambierdiscus* sp. type 3); S080911_1 (*G. australes*), were maintained in glass Petri dishes (SANSYO, Tokyo, Japan) filled with 20 mL of Daigo IMK medium under culturing conditions described as above. Cells in the late stationary phase (approx. 30 days) were harvested by filtration using 20 µm Nitex mesh and/or centrifuging. Toxic extracts were prepared by extraction from cell pellets, by using the method modified after Chinain [18], thrice in methanol (2 mL for a total biomass of 1.0×10^6^ cells) and thrice in aqueous methanol (MeOH:H_2_O 9∶1) (1 mL for a total biomass of 1.0 × 10^6^ cells) under sonication, for 15 min. each. After dividing toxic extracts into the required number of specimens, the extract was evaporated, a solvent partition was applied to the resulting reside using 0.4 mL of dichloromethane (CH_2_Cl_2_) once and 0.2 mL of aqueous methanol (MeOH:H_2_O 6∶4) twice. During liquid-liquid partitions, the dichloromethane and aqueous methanol phases, in which CTXs and MTXs are recovered respectively, were handled with extreme care in order to limit carry over of MTXs into the dichloromethane phase, and vice versa. The dichloromethane soluble fraction (DSF) and aqueous methanol soluble fraction (MSF) were evaporated, respectively.

The DSF and MSF of six strains of *Gambierdiscus* sp. were tested for their toxicity using mouse bioassay. Three or five mice (male, ddY, 20 g; Japan SLC, Inc, Shizuoka, Japan) were intraperitoneally administered with a single dose of toxic extract dissolved in 500 µl of a 0.85% saline solution containing 1% Tween 60. Mice were observed over 24 h and signs and time of death recorded. Fractions were considered non-toxic if injection of a maximal dose was not lethal. Total lethality was expressed in mouse units (MU) 1,000 cells^−1^, one MU is defined as the i.p. LD_50_ dose for a 20 g mouse over 24 h.

## Supporting Information

Figure S1 **Maximum likelihood (ML) phylogeny of the D8–D10 region of the LSU rDNA of** ***Gambierdiscus*** **species/phylotypes.** Nodal supports are of ML analysis. Nodes with strong supports (pp/bt  = 1.00/100) are shown as thick lines. For sequences obtained via cloning, a variant ID, starting with C, is shown followed by strain ID (i.e. T080908_1_C1). Sequences obtained in the present study are indicated in color.
(TIF)

Table S1 **Estimation of genetic distances of the SSU rDNA over the** ***p*** **distance calculated by uncorrected genetic distance (UGD) model among and within 69 consensus sequences ( = 69 strains; each consensus sequence was constructed with cloned sequences and/or directly sequence of each strain) of** ***Gambierdiscus*** **species/phylotypes.**
(XLS)

Table S2 **Summary of genetic distances of the D8–D10 region of the LSU rDNA over the** ***p*** **distance calculated by uncorrected genetic distance (UGD) model among and within 51 consensus sequences ( = 51 strains; each consensus sequence was constructed with cloned sequences and/or directly sequence of each strain) of** ***Gambierdiscus*** **species/phylotypes.**
(XLS)

Table S3 **Estimation of genetic distances of the D8–D10 region of the LSU rDNA over the** ***p*** **distance calculated by uncorrected genetic distance (UGD) model among and within 61 consensus sequences ( = 61 strains; each consensus sequence was constructed with cloned sequences and/or directly sequence of each strain) of** ***Gambierdiscus*** **species/phylotypes.**
(XLS)

Table S4 **Details of macroalgal sample collection.**
(XLS)

Table S5 **Details of** ***Gambierdiscus*** **spp. clonal strains.**
(XLS)

Table S6 **Details of toxicities of Japanese** ***Gambierdiscus*** **species/phylotypes tested by a mouse bioassay.** One MU is defined as the LD_50_ dose for a 20-g mouse over 24 h (n = 3 or 5 in the present study).
(XLS)
