# Supplementary figures and images for: Genetic Diversity and Distribution of the Ciguatera-Causing Dinoflagellate Gambierdiscus spp. (Dinophyceae) in Coastal Areas of Japan
Source: PLoS One. 2013 Apr 11;8(4):e60882. doi: 10.1371/journal.pone.0060882 (PMC3623954; doi:10.1371/journal.pone.0060882)

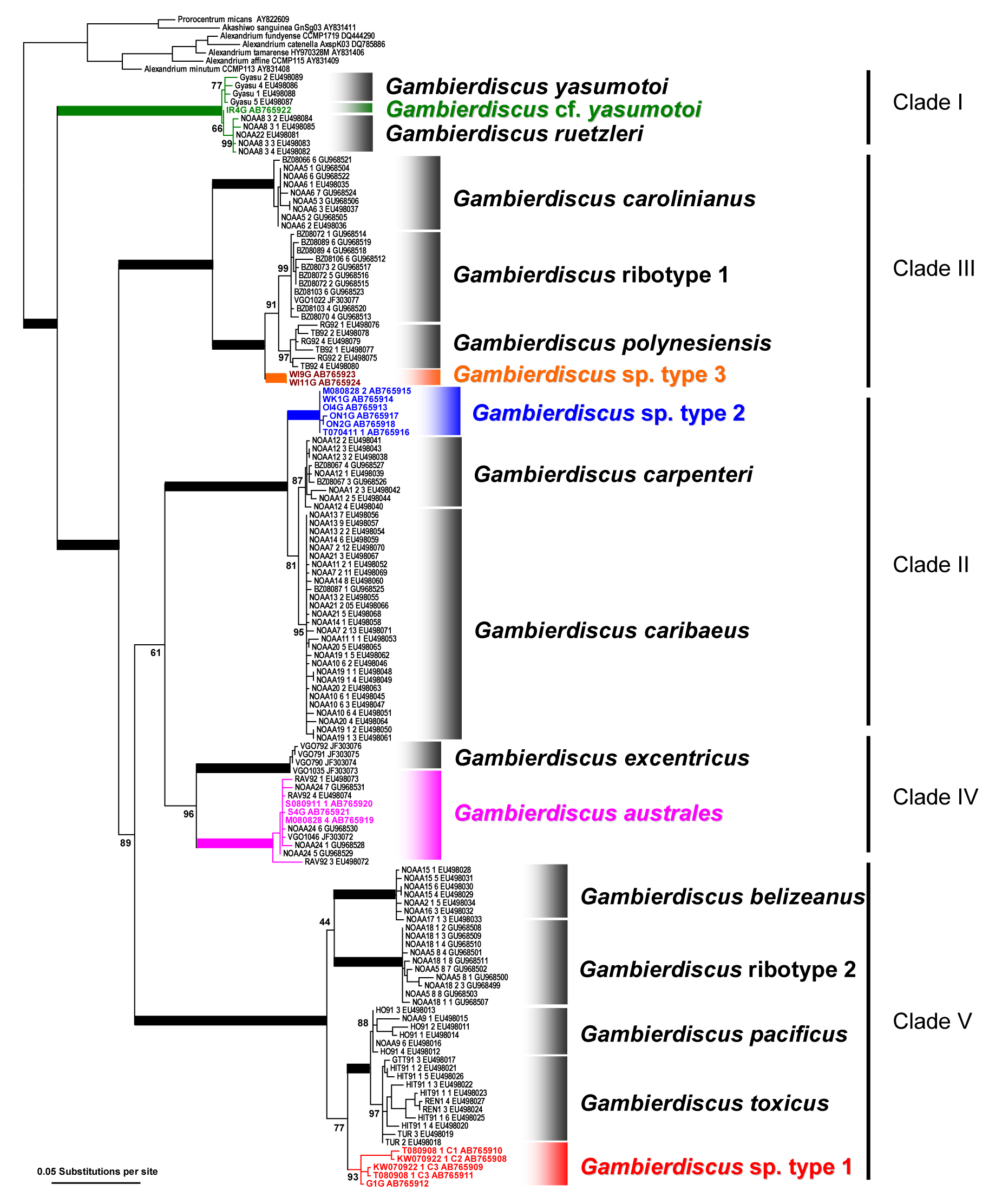

Supplement: Figure S1 — Maximum likelihood (ML) phylogeny of the D8–D10 region of the LSU rDNA of Gambierdiscus species/phylotypes. Nodal supports are of ML analysis. Nodes with strong supports (pp/bt = 1.00/100) are shown as thick lines. For sequences obtained via cloning, a variant ID, starting with C, is shown followed by strain ID (i.e. T080908_1_C1). Sequences obtained in the present study are indicated in color. (TIF) [file pone.0060882.s001.tif]
